# Altered Expression of Human Smooth Muscle Myosin Phosphatase Targeting (MYPT) Isovariants with Pregnancy and Labor

**DOI:** 10.1371/journal.pone.0164352

**Published:** 2016-10-31

**Authors:** Jon Lartey, Julie Taggart, Stephen Robson, Michael Taggart

**Affiliations:** 1 Institute of Cellular Medicine, William Leech Building, Medical School, Newcastle University, Framlington Place, Newcastle upon Tyne, United Kingdom, NE2 4HH; 2 Institute of Genetic Medicine, Newcastle University, International Centre for Life, Central Parkway, Newcastle upon Tyne, United Kingdom, NE1 3BZ; International Centre for Genetic Engineering and Biotechnology, ITALY

## Abstract

**Background:**

Myosin light-chain phosphatase is a trimeric protein that hydrolyses phosphorylated myosin II light chains (MYLII) to cause relaxation in smooth muscle cells including those of the uterus. A major component of the phosphatase is the myosin targeting subunit (MYPT), which directs a catalytic subunit to dephosphorylate MYLII. There are 5 main MYPT family members (MYPT1 (PPP1R12A), MYPT2 (PPP1R12B), MYPT3 (PPP1R16A), myosin binding subunit 85 MBS85 (PPP1R12C) and TIMAP (TGF-beta-inhibited membrane-associated protein (PPP1R16B)). Nitric oxide (NO)-mediated smooth muscle relaxation has in part been attributed to activation of the phosphatase by PKG binding to a leucine zipper (LZ) dimerization domain located at the carboxyl-terminus of PPP1R12A. In animal studies, alternative splicing of PPP1R12A can lead to the inclusion of a 31-nucleotide exonic segment that generates a LZ negative (LZ-) isovariant rendering the phosphatase less sensitive to NO vasodilators and alterations in PPP1R12ALZ- and LZ+ expression have been linked to phenotypic changes in smooth muscle function. Moreover, PPP1R12B and PPP1R12C, but not PPP1R16A or PPP1R16B, have the potential for LZ+/LZ- alternative splicing. Yet, by comparison to animal studies, the information on human MYPT genomic sequences/mRNA expressions is scant. As uterine smooth muscle undergoes substantial remodeling during pregnancy we were interested in establishing the patterns of expression of human MYPT isovariants during this process and also following labor onset as this could have important implications for determining successful pregnancy outcome.

**Objectives:**

We used cross-species genome alignment, to infer putative human sequences not available in the public domain, and isovariant-specific quantitative PCR, to analyse the expression of mRNA encoding putative LZ+ and LZ- forms of PPP1R12A, PPP1R12B and PPP1R12C as well as canonical PPP1R16A and PPP1R16B genes in human uterine smooth muscle from non-pregnant, pregnant and in-labor donors.

**Results:**

We found a reduction in the expression of PPP1R12A, PPP1R12BLZ+, PPP1R16A and PPP1R16B mRNA in late pregnancy (not-in-labor) relative to non-pregnancy. PPP1R12ALZ+ and PPP1R12ALZ- mRNA levels were similar in the non-pregnant and pregnant not in labor groups. There was a further reduction in the uterine expression of PPP1R12ALZ+, PPP1R12CLZ+ and PPP1R12ALZ- mRNA with labor relative to the pregnant not-in-labor group. PPP1R12A, PPP1R12BLZ+, PPP1R16A and PPP1R16B mRNA levels were invariant between the not in labor and in-labor groups.

**Conclusions:**

MYPT proteins are crucial determinants of smooth muscle function. Therefore, these alterations in human uterine smooth muscle MYPT isovariant expression during pregnancy and labor may be part of the important molecular physiological transition between uterine quiescence and activation.

## Introduction

The human uterus during pregnancy undergoes a dramatic increase in size whilst maintaining a state of relative quiescence to accommodate the growing fetus and placenta. These morphological and physiological changes culminate at the end of pregnancy in a switch to a coordinated active contractile state to facilitate delivery of the fetus and placenta. The mechanisms that regulate the effective transitioning from the pro-gestational quiescent phase into the contractile phenotype required for labor remain poorly understood but are likely to include alterations in myofilament contractile phenotype evinced by differences in sensitivity to physiological stimuli.

Contraction of uterine smooth muscle is regulated by action potential- and G-protein-coupled receptor (GPCR) agonist-generated increases in intracellular calcium leading to Ca^2+^- calmodulin- dependent myosin light chain kinase (MYLK) activation and phosphorylation of the regulatory light chains of myosin (MYLII). This facilitates acto-myosin crossbridge formation and force enhancement. Myosin phosphatase (MYLP) is a trimeric protein that reverses MYL II phosphorylation to cause smooth muscle relaxation. MYLP is made up of a 38 kDa catalytic type 1 protein phosphatase (PP1c) subunit which dephosphorylates MYII, a 110 kDa targeting subunit—called myosin phosphatase targeting subunit (MYPT)—which controls the activity of the PPP1c subunit and a small 20 kDa regulatory (M20) subunit [1]. In addition to the Ca^2+^-dependent activation of MYLK and force enhancement, the GPCR pathway inhibits myosin phosphatase activity leading to additional force enhancement at any given [Ca^2+^][2]. Nitric oxide (NO) donors activate MYLP to enhance smooth muscle relaxatory capacity. It has been suggested that, in part, this arises from protein kinase G (PKG) binding to a leucine zipper (LZ) region of the MYPT subunit resulting in enhanced activity of MYLP protein, myosin dephosphorylation and smooth muscle relaxation[3,4] [5].

**Fig 1:**
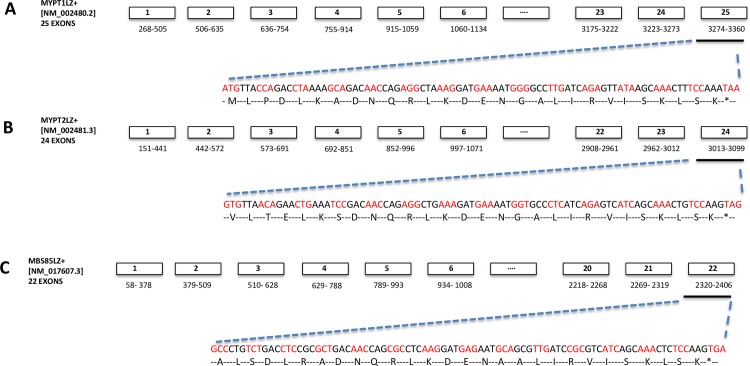
Schematic representation of the exon map of mammalian PPP1R12A, PPP1R12B and PPP1R12C leucine zipper positive (LZ+) isovariants. A-C. The NCBI accession numbers, nucleotide sequence identity and exon map of known MYPT LZ positive isovariants are shown. Nucleotide and amino acid sequences depicting the 4-heptad leucine repeat within the carboxyl-terminal exons are also displayed above.

There are five main mammalian MYPT related proteins each the product of separate genes: MYPT1 (also known as PPP12R1A (http://www.genenames.org/), MYPT2 (PPP1R12B), MYPT3 (PPP1R16A), MBS85 (PPP1R12C) and TIMAP (PPP1R16B). PPP1R12A, PPP1R12B and PPP1R12C are MYPT isoforms that have up to 80% amino acid sequence homology in the leucine zipper (LZ) dimerization region [X]L[XX]L[XX]DNQRLKDEN[X]ALIRVISKLSK (Fig 1). PPP1R16B and PPP1R16A are distantly related isoforms capable of auto-activation of PPP1c phosphatase function [6]. The significance of the LZ region (LZ+) of PPP1R12A, and possibly PPP1R12C and PPP1R12B, appears to reside in it conferring sensitivity to PKG binding and activation of the phosphatase [4,5]. However, alternative splicing of PPP1R12A results in the inclusion of a 31-nucleotide exonic segment that shifts the open reading frame to generate a shorter PPP1R12A isovariant that is LZ negative (LZ-) [7].The LZ- isovariant has been suggested to be less sensitive to NO donors or PKG binding[8]. Thus, the LZ- and LZ+ isovariants of PPP1R12A may influence different phosphatase activities. Further, their relative expressions are regulated in tissue and biological context-specific manners [9]. For example, large arteries with a slow contractile phenotype, like the aorta and pulmonary artery, show higher levels of the LZ+ isovariant and increased responsiveness to nitric oxide/PKG-mediated stimuli [10]. In contrast, smooth muscle tissues that have a fast contractile phenotype, such as the portal vein may express higher levels of the LZ- isovariant rendering them relatively insensitive to PKG-mediated relaxation [10]. Alterations in smooth muscle PPP1R12A LZ- to LZ+ expression have also been reported to be associated with developmental or phenotypic differences [8,11].

In human smooth muscle it is unknown if changes in PPP1R12ALZ+ and PPP1R12ALZ- isovariants occur with altered physiological circumstances. Indeed, the human PPP1R12A gene sequence [http://www.ncbi.nlm.nih.gov/nuccore/NM_002480.2] does not list a PPP1R12ALZ- isovariant. Similarly, only sequences encoding for LZ+ forms of PPP1R12C and PPP1R12B are listed. This leaves open the general question of if, and to what extent, LZ- isovariants of PPP1R12A, PPP1R12C and PPP1R12B are expressed in human smooth muscle. Furthermore it is not known how the expressions of the 5 canonical MYPT gene products, and the relative expressions of individual MYPT LZ+/LZ- isovariants in human uterine smooth muscle, vary with pregnancy and labor. There is some recent evidence pointing to alterations of PPP1R12A phosphorylation being associated with pregnancy [12] and with the uterine smooth muscle contraction-relaxation cycle [13] adding importance to the need to understand the breadth of expression of MYPT family members evident in these circumstances. We propose that the switch of human uterine smooth muscle from pro-gestational relative quiescent phenotype to a contractile phenotype may be associated with alterations in the expression of MYPT isoforms. Therefore, this study encompasses two approaches. First, we have performed cross-species bioinformatic analyses to infer the putative sequences of LZ- isovariants of human PPP1R12A, PPP1R12C and PPP1R12B. Second, using quantitative PCR, we have investigated the expression patterns of mRNA encoding known PPP1R12LZ+ and predicted PPP1R12LZ- isovariants, as well as PPP1R16A and PPP1R16B, in uterine tissue from non-pregnant women, term pregnant women not in labor and those in active labor.

## Materials and Methods

### Sample collection and tissue microdissection

All research involving human participants were approved by National Research Ethics Service Committee North East—Newcastle & North Tyneside 1 Research Ethics Committee reference: 08/H0906/21+5 and conducted according to the principles expressed in the Declaration of Helsinki. All donors gave written informed consent. Myometrial tissue was obtained from non-pregnant (NP) premenopausal women undergoing hysterectomy for benign gynaecological disorders (e.g. dysmenorrhea, menorrhagia) and from non-laboring pregnant women at term (37 to 42 weeks gestation) undergoing Cesarean delivery with the following indications: maternal request, breech presentation, previous Cesarean delivery or placenta previa. Samples were also obtained from women in active labor who had Cesarean deliveries for fetal distress, poor progress and maternal request. Myometrial biopsies were obtained from the upper part of the lower uterine segment hysterotomy for Cesarean section Patient’s details are outlined in Table 1. Myometrial biopsies were immediately placed in ice-cold tissue collection buffer (TCB); modified Krebs solution -154mM NaCl / 5.4mM KCl / 1.2mM MgSO_4_.7H_2_0 / 10mM MOPS / 5.5mM glucose / 1.6mM CaCl_2_.2H_2_0; pH 7.4) and transported to the laboratory where they were immediately micro-dissected and cleaned of all adherent fat and extraneous tissue, using a stereomicroscope. The micro-dissected segments were immediately frozen in liquid nitrogen and stored at -80°C until subsequently RNA extraction and quantitative real time PCR analysis.

**Table 1:** Details of donors used in this study.

Characteristics	NP	NIL	IL
**Number of patients**	14	15	12
**Age**	37.5±1.8	32±2.0	31.5±1.6
**Body mass index**	28±1.3	25.5±1.3	29±2.0
**Smoker**	36%	17%	0%
**Parity**	2.5±1.6	1±0.26	0.5±0.22
**Gravidity**	1±0.40	2.0±0.31	2.0±0.31
**Gestation age (weeks)**	41±0.40	39.0±0.34	41±0.63
**Birth weight (g)**		3365±147	3545±164
**Birth weight centile**		50±10.57	48±12.4

NP = non-pregnant; NIL = term not in labor; IL = in-labor.

### RNA and cDNA preparation

Micro-dissected myometrial tissues were homogenized and RNA extracted using a Qiagen RNeasy fibrous kit as previously described in [14]. Briefly, 30mg of tissue was homogenized in lysis buffer using a mechanical homogenizer (Minilys, Bertin Technologies). The tissue was homogenized for 5 cycles, each lasting 25 seconds and homogenate was kept on ice between cycles. Extracted RNA samples were treated with DNase I to digest any contaminant DNA and concentration and quality of the eluted samples were assessed by measuring optical density in a NanoDrop spectrophotometer (NanoDrop Technologies, Wilmington, DE, USA). RNA samples (100 ng) with A260/280 values > 1.8 and A260/230 values between 1.8–2.2 were subject to reverse transcription using AffinityScript Multiple Temperature cDNA synthesis Kit 200436 (Stratagene).

### Quantitative real-time PCR

Quantitative PCR (50 cycles) with PerfectProbe amplification (Primer Design, UK) was used to measure mRNA expression of individual MYPT isovariants using pre-verified primer sets (see below). The following protocol was used: enzyme activation hot start at 95°C for 10 min, denaturing at 95°C for 15 s and extension at 60°C for 60 s. The sample triplicates were within 1 Ct of each other and were >10 Ct values different from no template or water-only controls. No specific products were obtained in RT- and no template negative control reactions, indicating that all reagents were free of contamination and PCR products in the template positive reactions did not originate from genomic DNA contamination. 10 μl of sample cDNA, run in triplicate, were used as a template for each 25 μl PCR. Standard concentration-curves were generated using human reference smooth muscle cDNA (product # 636547, Clontech, France), which also served as an in-assay calibrator for fold-change in gene expression assessment.

PCR cycling conditions for individual primer sets, prior to PerfectProbe annealing, were optimized using SYBR Green amplicon detection in human smooth muscle reference RNA, human skeletal muscle RNA, cardiac smooth muscle and uterine smooth muscle RNA. SYBR Green cycling parameters were as follows: initial heat step at 95°C for 3 minutes, denaturing at 95°C for 15 s, annealing at 60°C for 60 s, extension at 72°C for 60 seconds followed step and hold dissociation curve step. Product amplicons from individual primer sets were cloned into a ‘gBlock gene fragments’ double stranded synthetic template and the resultant sequence verified positive control amplicon template was resuspended to obtain a working dilution of 2×10^5^/ μl. The amplicon template complex was run along with all the samples of interest as an internal positive control with a Ct of 18–20 indicating optimal cycling parameters for the primer in question.

### Agarose gel electrophoresis

The size of amplicon obtained from the reactions above was assessed using agarose gel electrophoresis. Briefly, 8 μl of PCR products was mixed with 2 μl of loading buffer (40% sucrose, 1 mM EDTA and 0.03% xylene cyanol) and separated by gel electrophoresis on a 2% agarose gel stained with ethidium bromide. The products were visualized using ultraviolet light transilluminator and photographed using Kodak camera according to the manufacturer’s instructions.

### Statistics

MYPT data sets were not to be normally distributed and therefore the data were log transformed using the formula [Y = log (Y)] and analysed using GraphPad Prism 4.0 (Hearne Scientific Software). Differences in the mean mRNA expression between non-pregnant and pregnant groups were assessed using ANOVA with Tukey’s Post hoc analysis. Results are presented as mean ± SEM and statistical significance was taken as p < 0.05.

## Results and Discussion

### Bioinformatic inference of human PPP1R12A, PPP1R12B and PPP1R12C LZ- sequences

**Fig 2:**
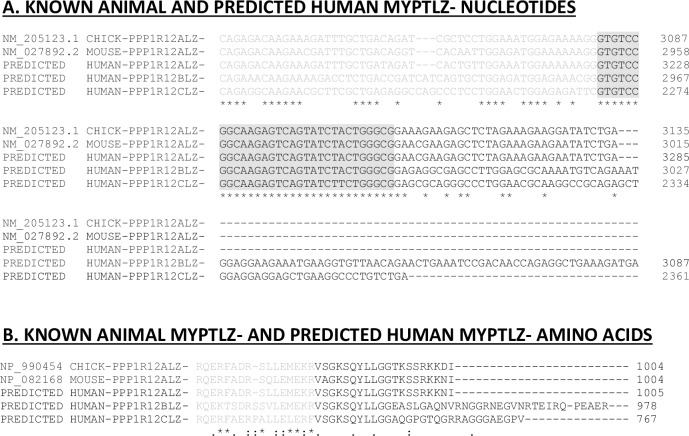
Bioinformatic inference of putative human PPP1R12A, PPP1R12B and PPP1R12C LZ- isovariant sequences. The publicly available sequence information for chicken and mouse PPP1R12ALZ- isovariants are shown to display the 31 nucleotide exonic insert ‘GTGTCCGGCAAGAGTCAGTATCTACTGGGCG’. This generates a shift in the reading frame and a LZ negative read is located at the start of the black lettered exon. Cross-alignment with the human published PPP1R12A, PPP1R12B and PPP1R12C sequences enabled identification of the region to theoretically insert the 31 nucleotides. Doing so resulted in the translated LZ negative protein sequence ‘V[X]GKSQYLLGG’ of the chicken and mouse sequences also appearing in the predicted human LZ- sequences.

**Fig 3:**
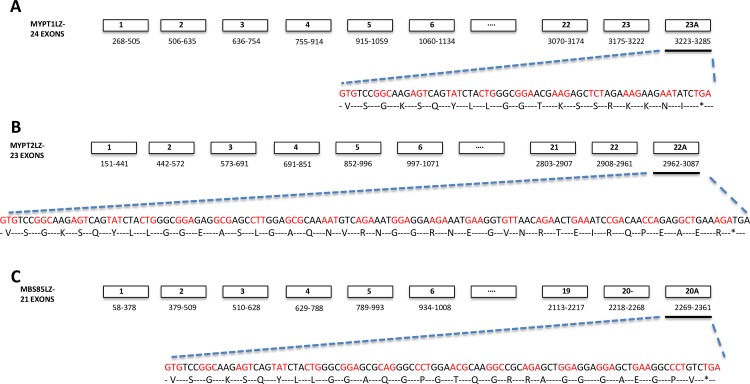
Exon map of ‘predicted’ human PPP1R12A, PPP1R12B and PPP1R12C LZ negative (LZ-) isovariants. The inferred LZ- exon maps, predicted amino acid and nucleotide sequences of the terminal exons of PPP1R12A, PPP1R12B and PPP1R12C (A-C) are displayed above.

Information on the LZ+ isovariants of human PPP1R12A [NM_002480.1], PPP1R12B (NM_002481.3) and PPP1R12C (NM_017607.3) sequences are publicly available sequence [http://www.ncbi.nlm.nih.gov/nuccore/NM_002481.3/NM_017607.3]. However, corresponding sequence information on the human LZ- isovariants is, surprisingly, lacking. Mammalian PPP1R12A LZ- sequences containing the 31-nucleotide exonic insert segment ‘GTGTCCGGCAAGAGTCAGTATCTTCTGGGCG’ are available for chicken (NM_205123) and mouse (NM_027892). Therefore, these were aligned to the human cDNA exonic sequences of PPP1R12A, PPP1R12B and PPP1R12C to enable species cross-comparison of these sequences. Figs 1–3 focus on the theoretical sequences of LZ+ and LZ- sequences in human MYPT thereby enabling the identification of isovariant-specific QPCR primers for various MYPTLZ+ and LZ- forms. Other investigators have presented similar multispecies bioinformatics analyses for the PPP1R12A gene [15]. This enabled identification of the C-terminal region at which generation of putative human LZ- isovariants could occur via insertion of a 31-nucleotide exonic segment (immediately prior to the last exon of the LZ+ versions). The resultant predicted human LZ- reads are displayed in Fig 3. The ‘spliced-in’ exon gene sequences, indicated as exon 23A of PPP1R12ALZ-, exon 22A of PPP1R12BLZ- and exon 20A of PPP1R12CLZ-, shifted the open reading frames to generate sequences that translate to amino acid sequences devoid of a LZ repeat (Fig 3).

**Fig 4:**
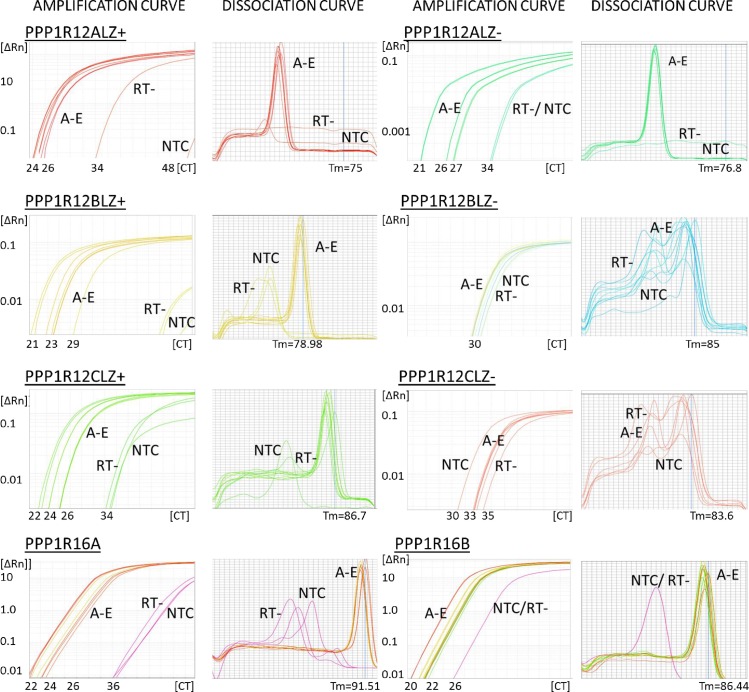
Optimisation of primers directed against PPP1R12A, PPP1R12B, PPP1R12C, PPP1R16A and PPP1R16B isovariants. Panels above show PCR amplification and dissociation curves obtained for PPP1R12ALZ+, PPP1R12ALZ-, PPP1R12BLZ+, PPP1R12BLZ-, PPP1R12CLZ+, PPP1R12CLZ-, PPP1R16A and PPP1R16B Curves labelled A-E are representative of specific product formation obtained from human smooth muscle reference cDNA and human uterine cDNA samples. Similar curves were obtained for other positive controls including human skeletal and cardiac muscle samples. No specific products were obtained in reactions where the RT enzyme was excluded (RT-) or in water no template control (NTC) reactions. No specific products were obtained with PPP1R12BLZ- or PPP1R12CLZ- primer sets.

Next, PCR primers were designed to either exclude (LZ+) or include the exonic segment (LZ-) of PPP1R12A, PPP1R12B or PPP1R12C to enable expression analysis individual LZ+ and LZ- isovariants. The sequences of individual primers and positions of primer-directed amplicons on the genes are displayed in Table 2 and S1 Table. Melt curve analyses of SYBR green QPCR reactions illustrated the formation of a single amplicon for PPP1R12ALZ+, PPP1R12ALZ-, PPP1R12BLZ+ and PPP1R12CLZ+, Fig 4. However, multiple primer sets for the PPP1R12BLZ- and PPP1R12CLZ- sequences failed to amplify PCR products with specificity (multiple peaks were observed in the melt curves) and/or insufficient divergence in amplification from no-template controls. This was suggestive of very low, or absent, transcript expression for PPP1R12BLZ- and PPP1R12CLZ- isovariants.

**Table 2:** Details of Primers used for quantitative PCR. The primers were based in the human cDNA sequences. The HUGO/GeneBank accession numbers, oligonucleotide sequences, position and amplicon size are outlined below and in S1 Table.

Isoform specificity of primers	Primer sequence	Position	GC content (%)	Predicted Melting Temperature (°C)	Product length
• PPP1R12ALZ+ • (NM_002480.2)	SENSE: 5′ TTGGAAATGGAAAAAAGGGAACG 3′	2936	39.1	76.8	120
	ANTISENSE: 5′ CCCATTTTCATCCTTTAGCCTCT 3′	3057	43.5		
• PPP1R12ALZ- • (NM_002480.2+31 NT INSERT)	SENSE: 5’ TGTCGGCAAGAGTCAGTAT 3’	2957	50	85	104
	ANTISENSE: 5’ TGTCGGCAAGAGTCAGTAT 3’	3060	37.5		
• PPP1R12A canonical • (NM_002480.2)	SENSE: 5’ AAGCACCACATCAACACCAA 3’	1704	45	60	192
	SENSE: 5’ ATGGTCACTGCCGTAGGAAC 3’	1895	55		
• PPP1R12BLZ+ • (NM_002481.3)	SENSE: 5’ GAGATGGAGAAACGGGAGAG 3’	2797	55	78.9	129
	ANTISENSE: 5’ TCTGATGAGGGCACCATTTT 3’	2925	45		
• PPP1R12BLZ- • (NM_002481.3+31 NT INSERT)	SENSE: 5’ TGTCCGGCAAGAGTCAGTAT 3’	2813	50	85	108
	ANTISENSE: 5’ GTGTCGGATTCAGTTCTGTTAA 3’	2919	37.5		
• PPP1R12B canonical • (NM_002481.3)	SENSE: 5’ TGAGAAGCCCACAGACACTG 3’	2025	55	60	227
	ANTISENSE: 5’ ATAGCAGGTGGGAACTGGTG 3’	2251	55		
• PPP1R12CLZ+ • (NM_017607.3)	SENSE: 5’ TGGAACTGGAGAGATTCGAGC 3’	2195	64	86.7	56.8
	ANTISENSE: 5’ TTGTCAGCGCGGAGGTC 3’	2288	52		
• PPP1R12CLZ- • (NM_017607.3+31 NT INSERT)	SENSE: 5’ CGCAGAGGCAAGAACGCT 3’	2138	64	83.5	87
	ANTISENSE: 5’ CGCCCAGTAGATACTGACTCTT 3’	2239	52		
• PPP1R16A • (NM_032902.5)	SENSE: 5’ TCTTCCCTCCCAGTGTTGTC 3’	669	55	91.5	200
	ANTISENSE: 5’ GTCACAGGCATTGATGTTGG 3’	862	50		
• PPP1R16B • (NM_015568.2)	SENSE: 5’ CGGACAGGACCAACCTGTAT 3’	1244	55	86.4	198
	ANTISENSE: 5’ CTCGTGGGATCTTGGTAGGA 3’	1422	55		
• MYH1 • (NM_005963)	SENSE: 5’AATCATAAGTGAAGAGTAATTTATCTAAC 3’	5896	24.1	56.4	95
	ANTISENSE: 5’ CATAAGTACAAAATGGAGTGACAAAG 3’	5990	34.6		
• ACTA2 • (NM_005963)	SENSE: 5’ TGACGAAGCACAGAGCAAAA 3’	171	45	56.4	128
	ANTISENSE: 5’ GGGCAACACGAAGCTCATT 3’	298	56.5		

PCR reactions indicated amplification of mRNA encoding PPP1R16A and PPP1R16B in the samples of interest. Therefore, primer sets for PPP1R12ALZ+, PPP1R12ALZ-, PPP1R12BLZ+, PPP1R12CLZ+, PPP1R16A and PPP1R16B were annealed to Perfect Probes to determine mRNA expression of each sequence in uterine smooth muscle samples from non-pregnant and pregnant not-in-labor (NIL) or in-labor (IL) women.

### Down regulation of PPP1R12ALZ+ and PPP1R12ALZ-, mRNA expression in labor

**Fig 5:**
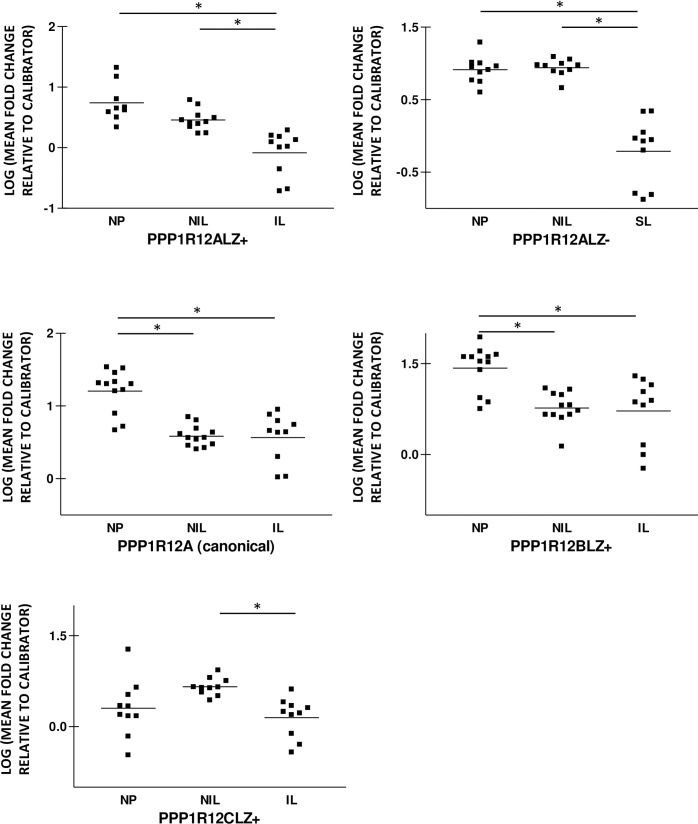
Human uterine PPP1R12ALZ+, PPP1R12CLZ+ and PPP1R12ALZ- mRNA expression is decreased during in labor. PPP1R12ALZ+, PPP1R12BLZ+, PPP1R12CLZ+ and PPP1R12ALZ- mRNA expression was determined using quantitative PCR and expressed as mean fold change relative to an internal calibrator. PCR amplification curves confirmed specific product formation for the four genes above. Quantitative PCR analyses demonstrate a significant decrease in PPP1R12ALZ+, PPP1R12ALZ- and PPP1R12CLZ+ mRNA expression in in-labor myometrium (IL) relative to pregnant not in labor (NIL) and non-pregnant (NP) myometrium. PPP1R12BLZ+ mRNA expression was lower in the IL and NIL groups relative to NP group. PPP1R12CLZ+ levels were similar NP and NIL groups. Bars represent means, *p<0.05, n = 10.

Isovariant-specific PerfectProbes detected amplification of mRNA encoding PPP1R12ALZ+ and PPP1R12ALZ- in all the non-pregnant and pregnant myometrial samples analysed in this study. PPP1R12ALZ+ mean fold-change relative to calibrator values was 0.74±0.11 in NP, 0.46±0.05 in NIL and 0.08.1±0.12 in the IL group. PPP1R12ALZ+ expression was decreased in the IL group relative to the NP and NIL groups, Fig 5. There was a reducing trend in PPP1R12ALZ+ expression in NIL compared to the NP group that failed to reach statistical significance. PPP1R12BLZ expression was 1.4±0.11-fold relative to control for NP, 0.77±0.08 for NIL and 0.72±0.17 in the IL group, consistent with a decrease in myometrial PPP1R12BLZ+ mRNA levels during pregnancy and in labor. PPP1R12BLZ+ mRNA expression was invariant between the NIL and IL groups. PPP1R12ALZ- mRNA expression was 0.91±0.06 for NP, 0.94±0.04 in NIL and 0.21±0.15 in the in-labor group demonstrating a significant decrease in PPP1R12A mRNA levels in the IL group compared to the NP and NIL groups. PPP1R12ALZ- expression was invariant between the NP and NIL groups.

In light of the differences in PPP1R12ALZ+ and LZ- expression outlined above, we also used primers aligned to exons upstream of the LZ region, which are common to both LZ+ and LZ- isovariants in order to detect the ‘canonical’ PPP1R12A mRNA expression in the three groups. PPP1R12A mRNA expression was 1.2±0.08-fold relative to control in NP, 0.59±0.04 in NIL and 0.57±0.11 in the IL group. Our analyses confirm a decrease in PPP1R12A mRNA expression in myometrium during pregnancy and in labor. Total PPP1R12A levels were similar in the NIL and IL groups, contrasting the significant reductions in PPP1R12ALZ+ and PPP1R12ALZ- mRNA expression in IL compared to NIL myometrium.

We also observed a decrease in PPP1R12CLZ+ mRNA levels in IL relative to NIL. PPP1R12CLZ expression was 0.30±0.15-fold relative to control in NP, 0.66±0.05 in NIL and 0.15±0.10 in the IL group. PPP1R12CLZ+ mRNA expression were invariant between the NP and NIL groups, Fig 5.

In summary, our results demonstrate a reduction in PPP1R12ALZ+, PPP1R12CLZ+ and PPP1R12ALZ- mRNA expression in human myometrium during labor. There was a decrease in the expression of PPP1R12BLZ+ during pregnancy and in labor.

### Expression of PPP1R16A and PPP1R16B mRNA in human myometrium during pregnancy and labor

**Fig 6:**
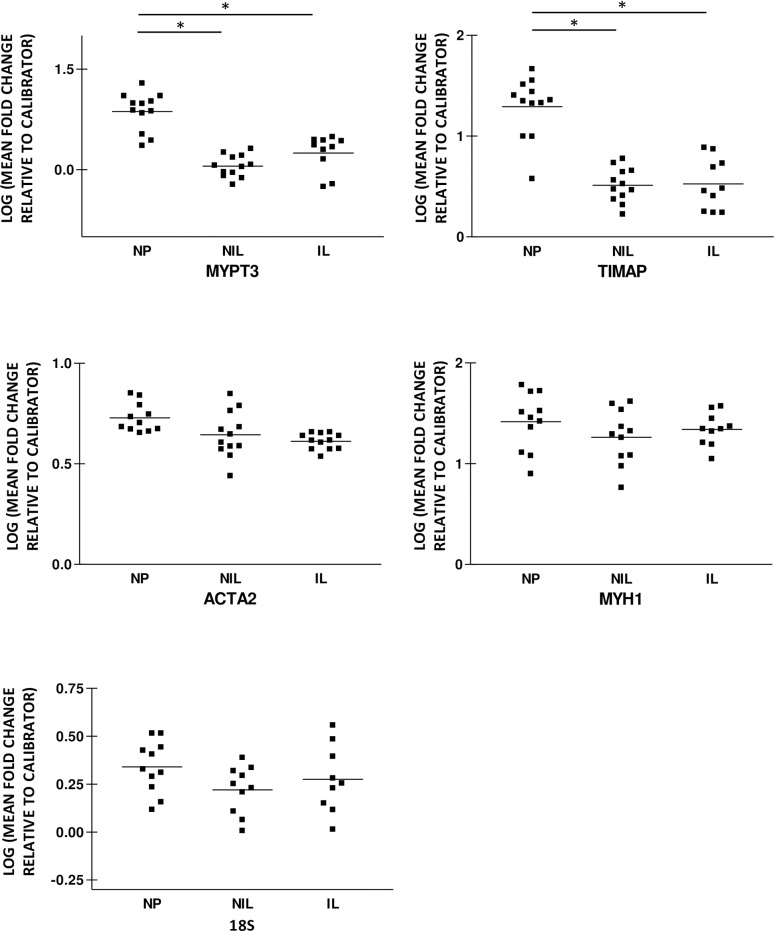
Human uterine PPP1R16A and PPP1R16B mRNA expression is decreased during pregnancy and in labor. PPP1R16A and PPP1R16B mRNA expression in non-pregnant (NP), pregnant not in labor (NIL) and in-labor (IL) myometrium were assessed using quantitative RT-PCR. The amount of individual MYPT mRNA in each sample was determined from human reference smooth muscle standard curve and quantified as mean fold change relative to an internal calibrator. PPP1R16A and PPP1R16B expression was significantly less in NIL and IL myometrium than in NP myometrium. PPP1R16A and PPP1R16B mRNA expression was similar in the NIL and IL groups. The bars represent mean, *p<0.05.

PPP1R16A and PPP1R16B mRNA expressions were reduced in the pregnant groups relative to the non-pregnant group, Fig 6. PPP1R16A mean fold changes were 0.87±0.08 relative to control in NP, 0.05±0.05 in NIL and 0.25±0.08 for the IL group. PPP1R16B expression was 1.29±0.09 in NP, 0.51±0.05 in NIL and 0.52±0.08 in IL consistent with a six-fold decrease in expression during pregnancy and in labor. PPP1R16A and PPP1R16B mRNA expression was invariant in between the pregnant NIL and IL groups.

We also assessed the expression of ribosomal RNA (18S) and two other smooth muscle related housekeeping genes myosin heavy chain 1 (MYH1) and alpha actin (ACTA2) in the non-pregnant and pregnant groups in order to determine if the changes above were either due to a down regulation in mRNA expression or as a consequence of pregnancy and labor induced exon splicing. MYH1, ACTA2, and 18S mRNA expression were similar in the non-pregnant, pregnant not in labor and in-labor groups, p>0.05. MYH1 and ACTA2 expression were 1.4±0.09 and 0.72±0.02-fold relative to control in NP, 1.3±0.07 and 0.64±0.03 in NIL and 1.3±0.08 and 0.61±0.01 in the IL group, Fig 6. 18S expression was 0.34±0.04 in NP, 0.18±0.06 in NIL and 0.28±0.06 for the IL group. This indicates that the down-regulation of most of the MYPT related genes during pregnancy and in labor are not as a consequence of a global down-regulation in smooth muscle related mRNA but rather due to alternative mechanisms including such as exon splicing. The reduction in canonical PPP1R12A levels with pregnancy is in part due to a reducing trend in PPP1R12ALZ+ mRNA expression in the pregnant not in labor compared to the non-pregnant groups. In contrast, the reduction in total PPP1R12A mRNA levels with labor is due to changes in both PPP1R12ALZ+ and PPP1R12ALZ- mRNA levels. It is plausible to conclude that these changes in PPP1R12ALZ+ and PPP1R12ALZ- mRNA levels with pregnancy and labor onset are isoform specific and may to be due to exon splicing.

In this study we investigated, for the first time in human smooth muscle, the pattern of expression of five canonical MYPT isovariants as well as exploring the possible existence of LZ- isovariants of human MYPT. Moreover, using human uterine smooth muscle samples from non-pregnant and pregnant patients, we show that there is a gestational-related change in expression of several MYPT isovariants. The possible functional implications of these findings for uterine function in pregnancy are discussed below.

Given the suggestions in the literature that variations in smooth muscle PPP1R12A LZ+ and PPP1R12ALZ- isovariants could impart functional consequences [10,16,17], it was surprising that, unlike the chicken and mouse genome, the human genome contains sequence information for PPP1R12ALZ+ but not PPP1R12ALZ- [7]. By aligning the chicken and mouse PPP1R12ALZ- sequences with the human PPP1R12ALZ+ sequences we could infer at which region of human PPP1R12A an insertion of the 31 nucleotide exonic segment, present in chicken and mouse, would likely arise–should there be a human PPP1R12ALZ- isovariant. Uniquely, we next inserted a similar putative insert in PPP1R12B and PPP1R12C to switch from LZ+ to LZ- reads. Subsequent QPCR experimentation indicated the existence of PPP1R12ALZ- expression in human uterine smooth muscle but we were unable to detect PPP1R12BLZ- or PPP1R12CLZ- sequences. This suggests that alternative splicing between LZ+/ LZ- isovariants of human MYPT in uterine smooth muscle primarily involves PPP1R12A. Our predicted PPP1R12ALZ- protein sequence was similar to PPP1R12ALZ- sequences from other species and another predicted human PPP1R12ALZ- sequence generated by a similar bioinformatic study[15].

Our results establish the presence of human PPP1R12ALZ- mRNA from a multi-tissue human smooth muscle reference homogenate (the commercially-used human reference RNA), cultured human uterine smooth muscle cells and human uterine smooth muscle tissue. We have previously reported PPP1R12ALZ+ and LZ- mRNA expression in human uterine and placental blood vessel tissues [14]. The PPP1R12ALZ- nucleotide and amino acid sequences generated are consistent with recently predicted PPP1R12ALZ- human sequences obtained from automated computational analysis using an NCBI eukaryotic gene prediction tool [http://www.ncbi.nlm.nih.gov/nuccore: PPP1R12ALZ- transcript variant X3: XM_011538376.1/ transcript variant X8: XM_005268887.2] and other bioinformatic analyses [15]. We are not aware of any reports of naturally occurring PPP1R12BLZ- and PPP1R12CLZ- isoforms at the time of this publication, but there are at least five ‘predicted’ PPP1R12B sequences [NP_001161329.1, NP_001161330.1, XP_011507877.1, XP_011507876.1, XP_011507875.1, XP_011507878.1 and XP_005245263.1] generated from computational analyses that lack the classic LZ motif.

We show that in late pregnancy there is down-regulation of myometrial PPP1R12A and PPP1R12BLZ+ mRNA relative to the non-pregnant state. PPP1R12ALZ+, PPP1R12CLZ+ and PPP1R12ALZ- mRNA levels were invariant between the non-pregnant and pregnant not in labor groups. Labor was associated with a reduction in the uterine expression of PPP1R12ALZ+, PPP1R12CLZ+ and PPP1R12ALZ- mRNA relative to the pregnant not-in-labor group Fig 5, PPP1R12A-C in S1 File. This is the first such report to show pregnancy-related changes in the differential myometrial expression of two other MYPTLZ+ genes namely pregnancy and in labor.

The reduction in expression of several MYPTLZ+ isovariants in late pregnancy and/or labor is consistent with molecular switches favoring an increased contractile phenotype (by virtue of a putative increased MYLII phosphorylation potential). In addition, a reduction in PPP1R12ALZ+ and PPP1R12CLZ+ isovariants with labor could confer reduced tissue sensitivity to PKG-mediated relaxatory influences. Of note, differing levels of PKG-induced Ca^2+^ desensitization in uterine and placental vasculature have been associated with changes in the expression of PPP1R12ALZ+ and other contractile associated proteins such as hsp20 and VASP[14]. However other workers have suggested that only small proportions of NO mediated relaxation in myometrium are actually mediated via PKG and cGMP related pathways, underlining the need for future investigations to explore alternative relaxatory pathways [18].

Other investigators have reported expression of PPP1R12A LZ+ and LZ- isoforms in both uterine and vascular smooth muscle in a range of animal models. However, findings in respect of MYPT expression during pregnancy are inconsistent. A 2-3-fold down-regulation of PPP1R12A mRNA expression in rat myometrium at the end of gestation has been reported by other investigators [19]. In contrast, Lontay and colleagues reported an increase in total MYPT mRNA and protein levels in mice and rat uterine smooth muscle and vascular tissues during pregnancy [20,21]. A review of other animal and human models has revealed ‘donor age’ as a potential confounder with remarkably contrasting effects on basal non-smooth and smooth muscle MYPT expression[21–23]. As there is no clear directional effect of age on MYPT expression it is therefore difficult to determine what impact, if any, the differences in mean age between the non-pregnant and pregnant donor groups have on the results above. Interestingly, the changes in PPP1R12ALZ+, PPP1R12CLZ+ and PPP1R12ALZ- mRNA expression with labor onset occurs between donor groups with a similar mean age.

MYPT2 (PPP1R12B) is expressed in the striated muscle and the brain [24]. It has similar sites of phosphorylation to PPP1R12A and an identical four-leucine heptad repeat in its carboxyl terminal region. In addition to its main function in dephosphorylating cardiac MYL, it has also been suggested to have roles in sarcomere organization during cardiac hypertrophy[25]. The reduced expression in late pregnancy does not exclude a role of PPP1R12B in regulating uterine cell hypertrophy in earlier stages of gestation, but such changes as we have observed are likely to represent a reduced overall phosphatase activity directed to phosphorylated MYL. In contrast to PPP1R12ALZ+, PPP1R12BLZ+ levels were similar in both the pregnant not in labor and in labor samples. The lack of evidence of PPP1R12BLZ- transcripts in our samples coupled with similarities in the expression patterns of the canonical PPP1R12B and PPP1R12BLZ+ isovariants in the three groups makes us suggest that previously noted exon splicing attributed to PPP1R12B by other authors is not present, or at least in a detectable amount according to our assays, in uterine smooth muscle at present[26]. We may need to use more primer sets in order to detect PPP1R12BLZ- in our samples.

Our results also demonstrate a 5-6-fold reduction in PPP1R16A and PPP1R16B expression in human myometrium during pregnancy with no further change in labor, Fig 6, PPP1R16A-B in S1 File. PPP1R16A and PPP1R16B are prenylatable subunits that inhibit PP1c activity towards phosphorylated myosin. Inhibitory phosphorylation of PPP1R16A by protein kinase A (PRKA) enhances PP1c activation with a resultant decrease in myosin phosphorylation and smooth muscle tone[6].

In light of our findings of a decrease in the expression of several uterine MYPT isovariants with pregnancy, it is reasonable to speculate that MTPT expression may be regulated by ovarian hormones estrogen and progesterone. In support of this, estradiol has also been shown to produce a 2-3-fold increase in aortic smooth PPP1R12A expression in a mice model of atherosclerosis[27]. Smoothelin-1 is also known to represses PPP1R12A expression, but pregnancy related changes on MYPT expression are similar in both wild type and smoothelin-deficient mice[21,28].

### Implications for the regulation of uterine function

Our results demonstrate a down regulation in mRNA expression of four MYPT isovariants in human uterine smooth muscle during pregnancy suggesting a reduced influence of phosphatase activity directed against the regulatory light chains of MYLII. This, in turn, may reflect a contribution of the regulation of MYPT expression to the shift in molecular physiological characteristics of the uterus from relative quiescence towards activation in preparation for parturition. That further reductions in PPP1R12A (LZ+ and LZ-) and PPP1R12CLZ+ mRNA occur in in-labor samples supports this notion. If the mRNA data were reflected at protein level, and MYLP activity, it would be indicative of a shift towards MYL phosphorylation and thereby increased uterine contractility.

## Supporting Information

S1 File PPP1R12A, PPP1R12B, PPP1R12C, PPP1R16A, PPP1R16B, MYH1, ACTA2 and 18S mRNA expression in non-pregnant and pregnant donors. (XLSX)

S1 Table Schematic representation of amplicon positions for PPP1R12A, PPP1R12B, PPP1R12C and PPP1R16A genes. The nucleotide positions (as indicated by asterisks) of human PPP1R12A, PPP1R12B and PPP1R12C LZ+ and LZ- primer sequences and the intended amplicons are displayed, as are those for PPP1R16A, PPP1R16B, MYH1 and ACTA2. The 31 nucleotide exonic insert, which generates LZ- read, is underlined for individual LZ- isovariants.
(DOCX)
