# Supplementary material for: Altered Expression of Human Smooth Muscle Myosin Phosphatase Targeting (MYPT) Isovariants with Pregnancy and Labor
Source: PLoS One. 2016 Oct 31;11(10):e0164352. doi: 10.1371/journal.pone.0164352 (PMC5087845; doi:10.1371/journal.pone.0164352)
Supplement: S1 Table — The nucleotide positions (as indicated by asterisks) of human PPP1R12A, PPP1R12B and PPP1R12C LZ+ and LZ- primer sequences and the intended amplicons are displayed, as are those for PPP1R16A, PPP1R16B, MYH1 and ACTA2. The 31 nucleotide exonic insert, which generates LZ- read, is underlined for individual LZ- isovariants. (DOCX) [file pone.0164352.s002.docx]

| PPP1R12ALZ+ sequence | ***********************  2936 TTGGAAATGGAAAAAAGGGAACGAAGAGCTCTAGAAAGAAGAATATCTGAAATGGAAGAA 2995  ***********************  2996 GAGCTCAAAATGTTACCAGACCTAAAAGCAGACAACCAGAGGCTAAAGGATGAAAATGGG 3057  3058 GCCTTGATCAGAGTTATAAGCAAACTTTCCAAATAA 3093 |
| --- | --- |
| PPP1R12ALZ- sequence | ********************  2945 TGGAAAAAAGGGTGTCCGGCAAGAGTCAGTATCTACTGGGCGGAACGAAGAGCTCTAGAA 3004  ************************  3005 AGAAGAATATCTGAAATGGAAGAAGAGCTCAAAATGTTACCAGACCTAAAAGCAGACAAC 3064  3065 CAGAGGCTAAAGGATGAAAATGGGGCCTTGATCAGAGTTATAAGCAAACTTTCCAAATAA 3124 |
| PPP1R12A canonical sequence | ********************  1681 GATTTGATTAGTTCTAGTGTTCCAAGCACCACATCAACACCAACAGTTACCTCTGCAGCT 1740  1741 GGGCTTCAGAAAAGCCTGCTTTCCAGCACAAGCACTACTACAAAGATTACAACGGGTTCT 1800  1801 TCCTCAGCAGGCACACAAAGCAGTACCTCAAATCGTTTGTGGGCTGAGGATAGTACTGAG 1860  ********************  1861 AAAGAAAAGGACAGTGTTCCTACGGCAGTGACCATTCCTGTTGCTCCAACTGTTGTAAAT 1920 |
| PPP1R12BLZ+ sequence | ********************  2796 GGAGATGGAGAAACGGGAGAGGCGAGCCTTGGAGCGCAAAATGTCAGAAATGGAGGAAGA 2855  2856 AATGAAGGTGTTAACAGAACTGAAATCCGACAACCAGAGGCTGAAAGATGAAAATGGTGC 2915  **********  2916 CCTCATCAGAGTCATCAGCAAACTGTCCAAGTAG 2949 |
| PPP1R12BLZ- sequence | ********************  2812 GTGTCCGGCAAGAGTCAGTATCTTCTGGGCGGAGAGGCGAGCCTTGGAGCGCAAAATGTC 2871  ***********************  2872 AGAAATGGAGGAAGAAATGAAGGTGTTAACAGAACTGAAATCCGACAACCAGAGGCTGAA 2931  2932 AGATGAAAATGGTGCCCTCATCAGAGTCATCAGCAAACTGTCCAAGTAG 2980 |
| PPP1R12B canonical sequence | ****************  1981 TTCAGCCGGTCGAGGGCAGAGAGGCAAGCTCAGGAGCAGCCTCGTGAGAAGCCCACAGAC 2040  ****  2041 ACTGAAGGGCTTGAGGGGAGCCCTGAGAAGCATGAGCCCTCAGCAGTTCCAGCAACAGAA 2100    2101 GCTGGGGAGGGCCAGCAGCCCTGGGGCAGGAGTCTGGATGAAGAGCCTATCTGTCATCGC 2160    2161 CTGAGGTGCCCAGCTCAGCCAGACAAACCCACCACGCCAGCATCTCCTTCTACGTCAAGA 2220  ********************  2221 CCCTCACTCTACACCAGTTCCCACCTGCTATGGACAAATAGATTTTCAGTCCCTGATTCT 2280 |
| PPP1R12CLZ+ sequence | *********************  2194 CTGGAACTGGAGAGATTCGAGCGCAGGGCCCTGGAACGCAAGGCCGCAGAGCTGGAGGAG 2253  **********************  2254 GAGCTGAAGGCCCTGTCTGACCTCCGCGCTGACAACCAGCGCCTCAAGGATGAGAATGCA 2313  2314 GCGTTGATCCGCGTCATCAGCAAACTCTCCAAGTGA 2349 |
| PPP1R12CLZ- sequence | ******************  2155 ACGCAGAGGCAAGAACGCTTCGCTGAGAGGCCAGCCCTCCTGGAACTGGAGAGATTCGTG 2211  **********************  2161 TCCGGCAAGAGTCAGTATCTTCTGGGCGGAGCGCAGGGCCCTGGAACGCAAGGCCGCAGA 2271  2272 GCTGGAGGAGGAGCTGAAGGCCCTGTCTGACCTCCGCGCTGACAACCAGCGCCTCAAGGA 2331  2332 TGAGAATGCAGCGTTGATCCGCGTCATCAGCAAACTCTCCAAGTGA 2380 |
| PPP1R16A sequence | ********************  661 GAAGCAGGTCCTCTTCCCTCCCAGTGTTGTCCTTCTGGAGGCCGCTGCCCGAAATGACCT 720  721 GGAAGAAGTCCGCCAGTTCCTTGGGAGTGGGGTCAGCCCTGACTTGGCCAACGAGGACGG 780  ********************  841 GGAGGCTGGGGCCAACATCAATGCCTGTGACAGTGAGTGCTGGACGCCTCTGCATGCTGC 900 |
| PPP1R16B sequence | ********************  1243 TCGGACAGGACCAACCTGTATAGGAAGGAGTATGAGGGAGAGGCCATCCTGTGGCAGCGG 1302  1303 AGTGCAGCTGAGGATCAGCGGACCTCCACCTACAACGGGGACATCAGGGAGACCAGGACA 1362  *  1363 GACCAAGAGAATAAGGACCCTAACCCCAGGCTGGAGAAGCCCGTGCTACTCTCCGAATTT 1422  *******************  1433 CCTACCAAGATCCCACGAG 1441 |
| MYH1 sequence | *****************************  5881 GGAGGTTCACACAAAAATCATAAGTGAAGAGTAATTTATCTAACTGCTGAAAGGTGACCA 5940  **************************  5941 AAGAAATGCACAAAATGTGAAAATCTTTGTCACTCCATTTTGTACTTATGACTTTTGGAG 6000  6001 ATAAAAAATTTATCTGCCAAAAAAAAAAAAAAAAAAAA 6038 |
| ACTA2 sequence | **********  121 AGACATCAGGGGGTGATGGTGGGAATGGGACAAAAAGACAGCTACGTGGGTGACGAAGCA 180  **********  181 CAGAGCAAAAGAGGAATCCTGACCCTGAAGTACCCGATAGAACATGGCATCATCACCAAC 240  *******************  241 TGGGACGACATGGAAAAGATCTGGCACCACTCTTTCTACAATGAGCTTCGTGTTGCCCCT 300 |
